# Ethical Dilemmas in Online Research and Treatment of Sexually Abused Adolescents

**DOI:** 10.2196/jmir.1455

**Published:** 2010-12-19

**Authors:** Alfred Lange, Jeroen Ruwaard

**Affiliations:** ^2^Interapy PLCAmsterdamNetherlands; ^1^Department of Clinical PsychologyUniversity of AmsterdamAmsterdamNetherlands

**Keywords:** Childhood abuse, adolescence, sexual abuse, codes of ethics, consent, anonymity, posttraumatic stress disorder, cognitive behavior therapy, cognitive behavior therapy methods, Internet, exposure, social sharing

## Abstract

**Background:**

In a recent uncontrolled trial of a new therapist-assisted Web-based treatment of adolescent victims of sexual abuse, the treatment effects were found to be promising. However, the study suffered a large pretreatment withdrawal rate that appeared to emanate from reluctance among the participants to disclose their identity and obtain their parents’ consent.

**Objective:**

Our objectives were to confirm the effects of the online treatment in a controlled trial and to evaluate measures to reduce pretreatment withdrawal in vulnerable populations including young victims of sexual abuse.

**Methods:**

The study was designed as a within-subject baseline-controlled trial. Effects of an 8-week attention-placebo intervention were contrasted with the effects of an 8-week treatment episode. Several measures were taken to reduce pretreatment dropout.

**Results:**

Pretreatment withdrawal was reduced but remained high (82/106, 77%). On the other hand, treatment dropout was low (4 out of 24 participants), and improvement during treatment showed significantly higher effects than during the attention placebo control period (net effect sizes between 0.5 and 1.6).

**Conclusions:**

In treatment of vulnerable young populations, caregivers and researchers will have to come to terms with high pretreatment withdrawal rates. Possible measures may reduce pretreatment withdrawal to some degree. Providing full anonymity is not a viable option since it is incompatible with the professional responsibility of the caregiver and restricts research possibilities.

## Introduction

More than a decade of research has shown that therapist-assisted Web-based treatment may provide an effective alternative to standard (face-to-face) treatment for a wide range of psychological disorders [1,2]. However, most of the evidence has been collected among adult populations. Further research is needed to establish the efficacy of such treatment for vulnerable children and adolescents.

In 2007, the Rutgers Nisso Group, a Dutch expert center on sexuality, initiated the development of a protocol for the online treatment of adolescent victims of sexual abuse and sexual violence. Dutch epidemiological research among 12 to 25 year old children, adolescents, and young adults had estimated the prevalence of sexual abuse to be 18% among girls and 4% among boys [3]. Other studies demonstrated that many victims do not disclose their experiences and that the accessibility of professional help was poor due to long waiting lists [4]. Clearly, there was a need for more accessible psychological help for this group. In their online project, Rutgers Nisso aimed to increase the availability of evidence-based care. They conjectured that adolescent victims would more readily seek online treatment, given their extensive use of the Internet and their tendency to disclose their feelings and thoughts more freely on the Internet [5].

Rutgers Nisso, the Interapy group—a Dutch center for research, development, and Internet treatment of psychological disorders—and the University of Amsterdam developed an online treatment based on an existing therapist-guided Web-based treatment of posttraumatic stress [6-10]. This protocol was adapted to victims of sexual abuse, and its effects were tested in an uncontrolled clinical trial. In that study, treatment dropout was low (all but one completed treatment), and the effects for those who started treatment were favorable. At posttreatment, participants reported substantial reductions on measures of posttraumatic stress and general psychopathology (.7 < d < 1.1). However, pretreatment withdrawal was very high (90%): only 8 of the 82 eligible applicants (10%) actually started treatment. Applicants withdrew in large numbers during the online screening prior to a diagnostic telephone interview [11]. Analyses of the pretreatment withdrawal suggested that the researchers’ obligation to ascertain parental consent and the supposed loss of anonymity for the participants discouraged many applicants from participating in the study.

The study raised several dilemmas:

Is it responsible to forgo biographical information that might be essential in the case of a personal crisis of the client? What is the responsibility of the care provider in that case? Obviously, the moral aspects seem to be the most compelling, but there may be financial consequences if claims of neglect are brought against the care provider. Legal questions may present themselves in countries such as the Netherlands where care providers are obliged to obtain and register the “Citizen Service Number” of all clients.

On the other hand, what are the ethical implications of withholding a promising treatment from the most vulnerable group?

How will outcome research suffer given the absence of the biographic information that is needed to conduct long-term follow-up, dropout analyses, and analyses of moderators of treatment effect?

Dilemmas associated with the requirement for parental consent and the loss of anonymity are not confined to treatment studies. In survey research, nonresponse increases considerably when anonymity is lifted, and informed consent is made obligatory [12]. After a general health examination with youngsters between 12 and 17 years of age, Lothen-Kline and colleagues [13] experimented with 2 exit questionnaires. The questionnaire informing the respondents that their data would be shared with parents or guardians showed significantly less affirmation regarding suicidal ideation and use of alcohol than the consent form that did not mention this. Some authors have discussed the age level up to which parents or guardians have to be informed. Some of them advocate lowering the age level because the cognitive development of youngsters is sufficient for them to decide themselves whether to participate [14]. The recommendations vary from “researchers should be responsible and know when to deviate from the normal age restrictions” to “researchers should adhere to the law with regard to the age of parental control” or “try to get dispensations.” However, issues of law and responsibility are often neglected as well. In a systematic review of 34 outcome studies regarding substance abuse, Smith et al found that in 59% the consent procedures were not reported adequately [15].

As noted by Childress [16], if the identity of a client cannot be verified, the caregiver runs the risk of treating minors without the knowledge and consent of their parents or guardians. Full anonymity does not seem to be a viable option in guided online treatment. Anonymous treatment may jeopardize the professional responsibility of the caregiver [17] and will restrict research possibilities. In general, two options seem feasible. First, one can reduce the anxiety about nonanonymity in the participants. This is especially important for potential clients who do not need parental consent but who nevertheless are hesitant to participate without strict anonymity. Second, if possible, one can change the designs of studies in such ways that parental consent might not be required.

In the next section, we present the design and outcome of a study that was conducted to obtain a controlled estimate of the effects of online treatment for young victims of sexual abuse. In this study, several measures were taken to reduce pretreatment withdrawal. The discussion reflects on the outcome for those who started treatment and the lessons that were learned with regard to pretreatment withdrawal.

## Methods

### Design

Several studies have demonstrated the effectiveness of the therapist-assisted Web-based treatment in adults [6-10]. A previous study confirmed these findings in an adolescent population [11]. Under Dutch law, in experimental (randomized) studies, parental consent is not needed for participants of 18 years and over. However, if the study is a nonrandomized evaluation of an existing treatment, this is lowered to 16 years and over. For that reason, the present study was designed as a treatment evaluation study, in a within-subject, baseline-controlled format. The baseline-control period consisted of a placebo intervention of 8 weeks comprising attention by providing fortnightly outcome measurements and encouraging messages. The treatment period followed and comprised 8 weeks of intervention, with 4 fortnightly outcome measurements. Since there was no randomization, participants who were 16 years or older did not need parental consent. The design was approved by the ethical committee of the Department of Psychology of the University of Amsterdam.

### Treatment

#### The Protocol

The treatment protocol was based on an existing cognitive behavioral treatment of posttraumatic stress in adult populations [6-10] and on previous research that suggested that victims of rape or other forms of sexual abuse often refrain from disclosing their experiences. In a large survey study, Lange et al [18] found that reactions to disclosure were critical in this association. Negative reactions, inducing shame and guilt, explained more of the variance in psychopathology than the “objective” severity of the abuse. The original treatment comprises 10 structured writing assignments [7,19] implementing 3 therapeutic modules: exposure, cognitive reappraisal, and social sharing.

Several changes were made to adapt the protocol to the treatment of victims of sexual abuse. First, an additional feedback occasion was included in the exposure module to provide extra guidance at this difficult stage [20,21]. Second, an extra module was added that comprised participants’ writing about the impact of the sexual abuse on their physical functioning, on their body image, and on their intimate relationships and sexuality. Third, at the end of treatment, instructions were added to generate a “personal toolkit,” that is, a document in which participants listed the treatment elements they found most useful. In this document, clients formulated how they would use these elements should they sense impending relapse. Finally, extra psycho-education was added concerning the specific problems participants might have encountered, such as shame, social anxiety, or lack of assertiveness. The treatment comprised 11 virtual contacts during the 8 weeks of treatment.

#### Setting

The full therapeutic procedure was conducted without face-to-face contact. Participants used a common Web browser to follow the procedure, including the completion of the questionnaires and the therapeutic assignments.

#### Privacy

Several measures were taken to secure the privacy of the participants. First, only the therapist and the participant were given access to the treatments. Participants and therapists were given an account to a private password-protected website. In addition, the website included a Web mail system, which allowed participants to contact their therapist outside the treatment regime. Thus, participants who shared an email account with others (eg, family members) did not have to use this shared account during treatment. Third, all communication with the website was encrypted with the Hypertext Transfer Protocol over Secure Socket Layer. Fourth, the Web server was protected by a firewall and remotely administered through an encrypted communication channel.

### Participants

#### Recruitment

In the previous study of treatment of victims of childhood sexual abuse [11], many applicants were excluded because they were older than 18 years of age. Later, strong indications from the field suggested that help was equally needed for young adults and for adolescents. Accordingly, the upper age level in the present study was raised to 25 years. Dutch media provided free publicity to the study in response to a press release. Potential clients were referred to a public website that provided background information about the study. This website contained an online application form.

#### Screening

The screening started with standardized self-report instruments administered through the secure website. To ease the fear of losing anonymity, the biographic questions (name, gender, telephone number, names of parents and general physician, and insurance data) were not posed at the beginning of the online screening, but in separate steps at later stages.

The online screening was followed by a diagnostic telephone interview conducted by graduated clinical psychologists. Applicants who were not willing to submit to the telephone interview were given the option of being interviewed through online text-based chat. To be included in the study, participants had to score at or above the clinical cutoff [24] for posttraumatic stress disorder (PTSD) on the Impact of Event Scale (IES) [22-24], described below. To establish whether the respondents experienced sexual abuse in the past, the Childhood Unwanted Sexual Experiences Questionnaire [25,26] was adapted for use with adolescents. It provides information about the type of the abuse, severity, feelings of guilt and shame, degree of disclosure, location, and the relationship with the perpetrator.

Risk of psychosis was determined by means of the Dutch Screening Device for Psychotic Disorder (SPDP) [27]. The Somatoform Dissociation Questionnaire-5 [28] was used to determine the degree of dissociation. Suicidal ideation was determined with the Dutch adaptation of the Suicidality Questionnaire [29]. The Dutch adaptation of the Self Harm Inventory [30] was used to establish the presence and degree of auto-mutilation. Applicants were excluded if they scored above the cutoff scores of these instruments. They were also excluded on grounds of any of the following: ongoing sexual abuse in the family; a prevalent disorder other than PTSD diagnosis; concurrent treatment; anorexia nervosa (body mass index [BMI] < 18); use of neuroleptica; prior admission into a psychiatric hospital; or substance abuse. Excluded respondents received personalized referrals to agencies providing face-to-face treatment in their region.

### Outcome Measures

#### The Impact of Event Scale

The Dutch adaptation of the IES was used to measure the degree of traumatization [22,23]. The IES consists of 15 items and comprises the subscales Intrusion (8 items) and Avoidance (7 items). Cronbach alpha varies between alpha = .66 and alpha = .78 for the Avoidance scale and between alpha =.72 and alpha = .81 for the Intrusion scale [31]. In the control period and during treatment, the IES was administered every 2 weeks.

#### Depression Subscale of the Symptom Checklist-90-Revised

To establish the degree of depression, the Dutch adaptation of the Depression subscale of the revised Symptom Checklist-90 (SCL-90-R) was used [32,33]. This scale comprises 16 items, which are scored on a 5-point Likert scale (0 to 4), indicating the rate of occurrence of depressive symptoms over the past week. The scale has good internal consistency (Cronbach alpha = .90) and good convergent and discriminant validity. The depression measure was administered 3 times: prebaseline, postbaseline/start treatment, and posttreatment.

#### Invalidation and Strength

Based on the methodology of Routine Outcome Monitoring [34], during the study, participants were repeatedly asked to express the degree to which their symptoms interfered with their functioning (ie, Invalidation) in the past week, on a scale from 1 (low) to 10 (high). Similarly, participants monitored their Strength, that is, the degree to which they had been able to cope with their symptoms in the past week. Correlations between Invalidation and Strength were calculated on all measurement moments. As to be expected, the correlations were negative, statistically significant (*P* = .005), and fairly high: the mean correlation was *r* = -.55 and ranged from *r* = -.30 to *r* = -.71. These associations became stronger in the second part of the study when the scores started to be affected by the therapeutic impact. These findings suggest that the measures, though associated, measure distinct constructs.

### Client Satisfaction

At posttest, participants answered questions regarding their satisfaction with the treatment in general and with its specific parts. In addition, they rated the therapeutic alliance, the nature of the online contact, whether they missed the face-to-face contact with their therapists, and their perceived effectiveness of the treatment.

### Statistical Analyses

Improvement was calculated for the baseline-control period and the treatment period separately. The differences between improvements during the control and treatment period were tested for each of the 4 outcome measures, using two-sided paired *t* tests. All participants, including those who did not complete the treatment, provided outcome data. Hence, the effects could be ascertained for all participants, including the dropouts (intention to treat), without statistical imputation techniques being necessary. The effect sizes were expressed in Cohen’s *d* [35] for both periods separately by dividing the mean improvement scores by the standard deviation of the first assessment. Net effect sizes were calculated by subtracting the effect size of the control period from the effect sizes of changes during the treatment period.

## Results

### Participant Flow

**Figure 1:**
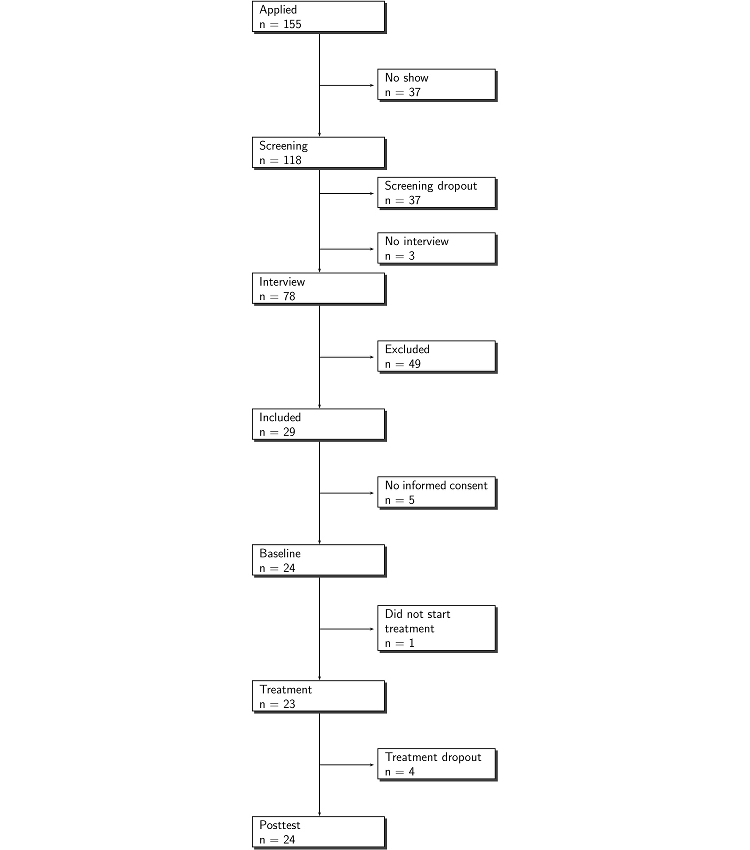
Flowchart of participation

Overall, as shown in Figure 1, this study also suffered from considerable pretreatment withdrawal. Of the 106 applicants that were not excluded by the researchers, 77 % (82) did not start the baseline-control period.

#### No Show

Of the 155 applicants, 24% (37) did not start the screening. Since we have no data for these respondents, we could not establish their age or their reasons for withdrawing.

#### Screening Withdrawal

In total, 118 applicants started the screening. Of these, 40 (34%) did not complete the screening. Most of the withdrawal (37 applicants) occurred during the online part of the screening. The online screening comprised 21 steps. Of these, 3 steps included biographic questions. Of those who withdrew during the online screening, 49% (18 out of 37) did so at one of these three steps.

Of the 81 applicants who completed the online screening, 3 did not commit themselves to the interview. Accordingly, 78 respondents were interviewed by telephone or chat. In total, 71 participants accepted the telephone interview, while 7 participants opted for the online chat.

#### Exclusion

Of the participants who completed the screening and were interviewed, 63% (49/78) met the criteria for study exclusion. The main reasons for exclusion were ongoing abuse within the family (n = 20) or being in concurrent treatment (n = 9).

#### Informed Consent

Of the 29 participations who were admitted to the study, 24 returned the completed informed consent form and were subsequently registered for participation in the treatment; 5 did not return the informed consent form. There was no difference in withdrawal percentage at this stage between those who had committed themselves to a telephone or chat interview.

#### Treatment Dropout

Of the 24 starting participants, 1 withdrew after the baseline-control period, and 4 dropped out during the treatment phase. All 24 starting participants completed the posttest.

### Age and Withdrawal

Table 1 presents the various forms of pretreatment withdrawal (screening dropout, refusing interview or chat, no informed consent) in different age groups. The table indicates that the younger groups showed higher rates of withdrawal than the older ones. Of the 65 applicants who had provided information about their age and were not excluded, the withdrawal was highest (7/8 or 87%) among the age group 14 to 15 years. The group aged 16 to 17 years old showed a withdrawal rate of 75% (12/16), whereas in the oldest group, 22 out of 41 (54%) withdrew before treatment started.

**Table 1:** Type of withdrawal by age group of applicants who were not excluded

	Age Group					
	14 -15 (n = 8)		16-17 (n = 16)		≥ 18 (n = 41)	
Type of Withdrawal	n	%	n	%	n	%
Screening dropout	5	62%	8	50%	20	49%
No interview	2	25%	1	6%	0	0%
No consent	0	0%	3	19%	2	5%
Total withdrawal	7	87%	12	75%	22	54%
Started baseline	1	12%	4	25%	19	46%

### Effects of Treatment

#### Baseline Characteristics

On average, participants who started treatment were 20 years old (range 14-25, SD 3.5). One participant was younger than 16, four were between 16 and 17 years old, and 19 participants were between 18 and 25 years old. An average of 5 years had passed (SD 4) since the occurrence of the traumatic events.

#### Outcome

Table 2 presents the averages of the participants on the outcome measures at screening, postcontrol/pretreatment, and at the end of treatment. The table shows large effect sizes for decrease in Invalidation and increase of Strength during treatment, while there were no or only small improvements during the control period. Accordingly, Table 2 shows large *net* effect sizes (difference in effect size between treatment and control) as well for Invalidation, Depression, and Strength. However, the net effect sizes on trauma symptoms as measured by the IES were moderate (*d* = .5) and not significant (*P* = .28).

**Table 2:** Means and standard deviations of outcome measures administered at the screening, at postcontrol period, and at posttreatment, effect sizes d, and t values resulting from the paired t tests of the differences in improvement during control and during treatment

	Screening/ Precontrol n = 24		Postcontrol/ Pretreatment n = 24			Posttreatment n = 24			Test of Difference	
Measure	Mean	SD	Mean	SD	*d*	Mean	SD	*d*	*t*_23_	*P*
IES^a^	49.5	9.1	35.7	15.2	1.5	17.5	15.4	2.0	1.1	.28
DEP^b^	43.7	13.3	41.8	12.0	.1	29.3	11.6	.9	3.3	.01
Invalidation	7.0	1.4	6.5	1.1	.4	3.6	1.7	2.0	3.7	.01
Strength	5.2	1.7	5.2	1.6	.0	7.1	1.6	1.1	3.2	.01

^a^ IES = Impact of Event Scale

^b^ DEP = Depression subscale of the revised Symptom Checklist-90

Overall, from screening to posttreatment, the effect sizes were very high, with Cohen’s *d* varying from *d* = 1.1 (strength) to 3.5 (trauma symptoms). Regarding the IES, all participants improved. According to the criteria of Jacobson and Truax [36], of the 24 participants, 2 (8%) changed only marginally, 5 (21%) reported reliable improvement, and 17 (71%) reported scores reflecting a reliable and clinically significant improvement.

#### Repeated Assessments

**Figure 2:**
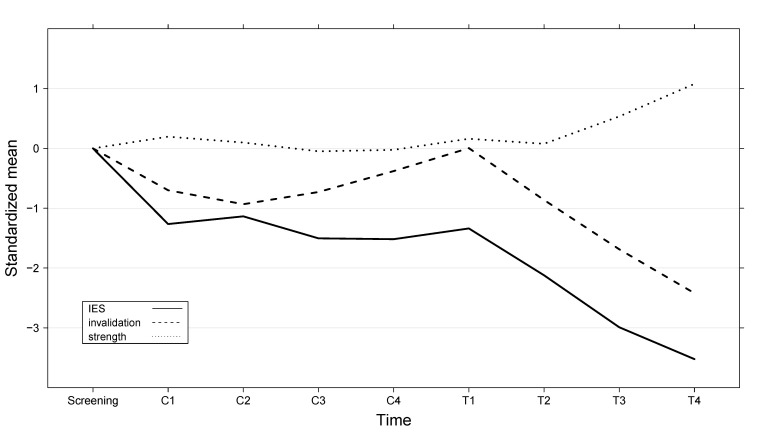
Standardized mean change in Impact of Event Scale (IES) scores and single-item assessments of Invalidation and Strength as measured weekly during the screening, the baseline control period (C1-C4), and the treatment period (T1-T4)

Figure 2 shows the process of change during control and treatment by the results of the fortnightly measurements of traumatic stress (IES), Invalidation and Strength. The figure displays the development of the standardized means over time: mean pretest scores were subtracted from the mean score at each measurement occasion and divided by the pretest standard deviation.

Figure 2 suggests that the large reduction in IES scores in the control period should be attributed to the screening. The screening included many questions that required the participants to focus on their trauma and on their present situations. In combination with the psycho-education and the expectation of the forthcoming treatment, this might have resulted in increases of awareness and hope. This ad hoc explanation is supported by Figure 2. The drop in IES right after the screening is very steep (this decrease represents an effect size of *d* = 1.3). After this, there is no further decrease in the IES scores during the baseline-control. When treatment started, the decrease started again, and persisted, during the whole treatment period.

In exploratory analyses, the change in trauma symptoms during the baseline-control period was again compared with the change during treatment. This time, the IES improvement scores were not calculated on the basis of the measurements taken during the screening but on the basis of the measurements taken at the start of the baseline-control, that is, at C1, the first measurement during the baseline-control period. This resulted in a significant difference between improvements in IES Scores made during treatment and the baseline-control period of *P* < .001, with a net effect of *d* = 1.8.

#### Client Satisfaction

As shown in Table 3, participants expressed general satisfaction with the treatment and their therapists. Although 22% of the participants did miss face-to-face contact, they were highly satisfied with their therapists, and 91% (21/23) stated that they would recommend the treatment to others. Treatment modules were evaluated favorably, in particular the exposure part of the writing. The new “Body” module, targeting bodily symptoms, received the lowest rating.

**Table 3:** Client satisfaction with treatment and therapists

Aspect		Response n = 23
**Satisfaction with Treatment (scale of 1 to 10)**		
	Overall, mean (SD)	7.9 (1.3)
	Writing/exposure phase, mean (SD)	8.1 (1.3)
	Body phase, mean (SD)	6.7 (2.1)
	Cognitive reappraisal phase, mean (SD)	7.3 (2.5)
	Taking leave/social sharing phase, mean (SD)	6.8 (2.9)
	Satisfaction with therapist , mean (SD)	8.6 (1.0)
Missed face-to-face contact, n (%)		5 (22.2%)
Internet therapy is an effective method, n (%)		20 (87%)
Would recommend the treatment to others, n(%)		21 (91%)

## Discussion

The first part of the discussion focuses on the outcome of the controlled study for those participants who started treatment. The second part focuses on the pretreatment withdrawal. Finally, we formulate on the basis of our results a set of recommendations regarding the ethical dilemmas concerning the online research into the treatment of young and vulnerable populations.

### Effects of Treatment

The data showed strong decreases in posttraumatic stress symptoms, depression, and subjective invalidation, and a strong increase in subjective strength. The tests between improvements in the baseline period and the treatment period were highly significant. The graphs of Invalidation and Strength showed gradual improvements that started after the first module and continued until the end of treatment.

At screening, the average IES score was well above the cutoff score for PTSD, and, at final posttest, the IES score was clearly below the cutoff score. From prebaseline (screening) to posttreatment, reductions in symptoms were significant and very large in terms of effects sizes. Taking these results into account, it is worth considering incorporating the screening and baseline period into the treatment itself. In future randomized trials, the effects of treatment with or without this baseline period should be investigated.

Ratings of the modules were generally high. Surprisingly, the lowest rating was given to the module that was specially generated for this population, psycho-education on somatic symptoms that might occur after sexual abuse. This might have been caused by specific frightening parts of the module comprising monitoring of behaviors including self-harm, obsessive cleanliness, and fear of being touched. Nevertheless, as shown in Figure 2, the relatively low satisfaction with this module did not interrupt the gradual improvements; there are indications that the module might even have given positive incentives to the next module of cognitive reappraisal.

The study is characterized by several strengths. In most experimental studies of the treatment of posttraumatic stress, the measures of the effects are expressed in terms of decrease in trauma symptoms. The present study confirms that treatment effect may also be expressed in the increase in feelings of strength. Our findings support the general suggestion to care providers and researchers to not focus entirely on the reduction of illness behavior, but to also target increase in self-esteem and empowerment [37,38]. Finally, the encouraging messages and repeated measures rendered the control period an attention placebo condition.

The content of the intervention was well established in prior research [6-10] and adapted to this special population in collaboration with an institution that is specialized in treating sexual problems in adults and adolescents. The protocol included many motivational techniques that inspired clients and therapists in bringing about a positive bonding [39-41]. The manner in which the online protocol was implemented allowed for strict control of treatment integrity.

Of course, this study also had its limitations, in addition to the considerable pretreatment withdrawal. First, only one male participated. The underrepresentation of males may be due to the greater incidence of sexual abuse among women. Also, a greater fear of disclosure in male victims may discourage them from seeking treatment [42,43]. We will have to find ways to encourage victimized male adolescents to seek evidence-based help. A second limitation is the absence (at the time of writing this report) of follow-up measurements. The follow-up measures will be ascertained up to one year after the posttest.

### Pretreatment Withdrawal

Of the 78 participants who completed all steps in the screening, 49 (62%) had to be excluded, a large proportion of them because the abuse was ongoing within the family or because they were already in treatment elsewhere. This demonstrates the vulnerability of this population.

In the previous study [11], many eligible applicants withdrew before treatment. In the present study, several measures were taken to reduce pretreatment withdrawal. First, the study was designed as an evaluation of treatment rather than an experimental randomized study. In this design, parental consent was obligatory only for applicants under 16 years of age instead of 18 years. Second, the upper age level for participation in the study was increased from 18 to 25 years. Thus, the population of potential participants who did not require parental consent was expanded. Third, participants were offered the alternative of a structured interview by chat if they were reticent to answer questions on the telephone.

The previous study showed a withdrawal rate of 90%. In the present study, the pretreatment withdrawal rate was 77%, a reduction of 13%: a total of 82 out of 106 applicants, who were not excluded by researchers, withdrew before treatment, while 24 (23%) started treatment. The present withdrawal rate is still high, but we should keep in mind that online treatment studies involving less sensitive populations also show considerable pretreatment withdrawal, varying from 19% to 46%, with an average of 37% [6-10].

The procedures during the screening permitted us to inspect at what stages withdrawal occurred. This inspection revealed that screening withdrawal was strongly associated with the posing of biographic questions. This again suggests that anonymity is probably the decisive factor, especially since the older participants—who did not need parental consent—also withdrew in high numbers when the biographic questions came up. We also learned that the youngest group (aged 14 to 15), who needed parental consent, withdrew nearly totally. Only 1 of the eligible applicants of that group started treatment (6%). The group of 16 to 17 years old did slightly better; 4 (25%) started treatment. The lowest pretreatment withdrawal was found in the oldest group, of which 46% of the eligible applicants started treatment.

Of course, caution is warranted in inspecting these results as they are based on relatively small numbers. But altogether, the data suggest that fear of losing anonymity is important for both young and old participants, whereas the fear of needing parental consent is more or less decisive for the younger age groups. Arranging the study as a treatment evaluation probably permitted the 16 to 18 years olds to participate in somewhat higher numbers since they did not need parental consent. The relatively low withdrawal in the oldest group supports this reasoning as well.

### Conclusion and Possible Approaches

Although pretreatment withdrawal occurs in most online treatments, it is worrisome that we are at present unable to reach a greater number of potential participants in the present type of vulnerable population. The measures taken to reduce pretreatment withdrawal seemed to have had some effects, but they were modest. Providing full anonymity is not an option since it is contrary to the professional responsibility of the caregiver, does not allow payment by insurance companies, and restricts research possibilities. However, high pre-treatment dropout should not discourage efforts to treat vulnerable groups. After all, the present study also revealed good adherence: having started the online treatment, few participants dropped out. Furthermore, in the treated group, the positive effects were large. The present procedures and findings should motivate us to find more effective ways of lowering the participation threshold without relaxing the clinical and scientific standards to which we subscribe. Even without the guarantee of anonymity, the following measures may reduce pretreatment withdrawal in so far as it is caused by fear of nonanonymity and the obligation of parental consent.

#### Loss of Anonymity

Determine which information is minimally necessary to carry out responsible interventions, for example, age, name, insurance details. Providers of health interventions could confine themselves to this minimally necessary information.

Provide information on the homepage about the necessity of gathering these biographic data. The most effective phrasing and timing of this information is an important issue that requires careful consideration.

During the screening, biographic questions should be preceded by an explanation of why each question is asked, and why the answer is optional or obligatory.

If biographic data are asked for scientific reasons only, make sure that clients are informed why the questions are asked and do not oblige them to answer those questions.

Increase the participant’s feeling of anonymity. This is especially important for those potential clients who do not need parental consent but are nevertheless reluctant to participate if anonymity is not guaranteed. Anonymity could be enhanced by posing fewer biographical questions. Information concerning actual identity could be requested at later stages in the program.

If parental consent is not needed, make sure that clients are informed that data are not shared with others.

#### Parental Consent

Seek dispensation regarding parental consent. For example, dispensation could be made conditional on the client’s disclosure to specially trained general practitioners [44]. Consent of one of these should then be sufficient to initiate the screening and ultimately start the online treatment. Note that in most countries, this would require a change in the law. Mental health institutions, political, and governmental institutions would have to make a concerted effort to realize the necessary changes to the law.

In countries in which the obligation of parental consent is stricter in research than in evaluation of treatment, one may facilitate participation by designing the study so that it, in effect, satisfies the definition of treatment evaluation.

If possible, consider changing the format from treatment to self-help. The present study comprised a full-fledged therapist-guided online treatment. Completely automated self-help programs might raise less anxiety about loss of anonymity. Many of the content protocols, such as those presented here, could be used in self-help programs. Yet, this option may still leave unresolved some legal and responsibility problems. Furthermore, there is growing evidence that the effects of pure self-help are different from the effects of guided self-help or online treatment [1,45,46].

### Final Remarks

We may simply have to accept that even when all measures described above are taken, the chances of encountering relatively high pretreatment withdrawal will remain considerable. Future studies should address this problem and describe the measures that were taken to reduce pretreatment withdrawal and the rates of withdrawal at various stages.

## Abbreviations

PTSD: posttraumatic stress disorder
IES: Impact of Event Scale
SPDP: Screening Device for Psychotic Disorder
BMI: body mass index
SCL-90-R DEP: Depression subscale of the revised Symptom Checklist 90
